# Emergency preparedness: What is the future?

**DOI:** 10.1017/ash.2021.190

**Published:** 2021-10-13

**Authors:** Jocelyn J. Herstein, Michelle M. Schwedhelm, Angela Vasa, Paul D. Biddinger, Angela L. Hewlett

**Affiliations:** 1 Department of Environmental, Agricultural, and Occupational Health, College of Public Health, University of Nebraska Medical Center, Omaha, Nebraska; 2 Nebraska Medicine, Omaha, Nebraska; 3 Department of Emergency Medicine, Massachusetts General Hospital, Boston, Massachusetts; 4 Division of Infectious Diseases, Department of Internal Medicine, College of Medicine, University of Nebraska Medical Center, Omaha, Nebraska

## Abstract

Emergency preparedness programs have evolved over the last several decades as communities have responded to natural, intentional, and accidental disasters. This evolution has resulted in a comprehensive all-hazards approach centered around 4 fundamental phases spanning the entire disaster life cycle: mitigation, preparedness, response, and recovery. Increasing frequency of outbreaks and epidemics of emerging and reemerging infectious diseases in the last decade has emphasized the significance of healthcare emergency preparedness programs, but the coronavirus disease 2019 (COVID-19) pandemic has tested healthcare facilities’ emergency plans and exposed vulnerabilities in healthcare emergency preparedness on a scale unexperienced in recent history. We review the 4 phases of emergency management and explore the lessons to be learned from recent events in enhancing health systems capabilities and capacities to mitigate, prepare for, respond to, and recover from biological threats or events, whether it be a pandemic or a single case of an unknown infectious disease. A recurring cycle of assessing, planning, training, exercising, and revising is vital to maintaining healthcare system preparedness, even in absence of an immediate, high probability threat. Healthcare epidemiologists and infection preventionists must play a pivotal role in incorporating lessons learned from the pandemic into emergency preparedness programs and building more robust preparedness plans.

Emergency preparedness programs have evolved over the last several decades as communities have responded to natural, intentional, and accidental disasters. This evolution has resulted in a comprehensive all-hazards approach centered around 4 fundamental phases spanning the entire disaster life cycle: mitigating the likelihood or reducing severity of hazards, preparing plans to improve capability and capacity to manage an emergency before it occurs, responding safely and effectively, and recovering from the event.^1^ These 4 phases, which represent a fluid and recurring cycle, are operationalized in the United States through the National Incident Management System framework, which must be adopted into organizational emergency management programs of hospitals and healthcare systems.^2^ This approach depends on improving coordination and cooperation among diverse entities involved in addressing and responding to all probable hazards, including biological threats such as a case of a high-consequence infectious disease (HCID), a bioterrorism event, or a global pandemic.

Following US terror attacks in the early 2000s, healthcare systems began to be viewed as a national security priority.^3,4^ Healthcare facility readiness to respond to community needs during a disaster has direct implications on the severity of the incident: mortality and morbidity associated with the event are also dependent on a facility’s preparedness and capabilities to effectively respond to and provide appropriate care for those affected. Beyond the health systems’ effective response, societal business continuity planning can inform prioritized strategies to minimize impacts to the economy, critical infrastructure, and resource acquisition.

Emergency preparedness in healthcare is inherently collaborative and requires a multidisciplinary approach, engaging a wide range of stakeholders in all phases of the disaster life cycle, including but not limited to, governmental agencies at all levels (local, state, and national), hospitals and other healthcare institutions, private businesses, and individual citizens. Within healthcare institutions, hospital epidemiologists, infection preventionists, and emergency managers are poised to play a pivotal role in infectious disease emergency management using their expertise in infectious diseases, infection prevention and control, and epidemiology; indeed, the critical nature of these roles was underscored during the COVID-19 pandemic. As retrospective analyses are completed and strategies from pandemic lessons learned are implemented, responsibilities of these professionals will continue to evolve and expand. Their unique qualifications and expertise position them as leaders in hospital pandemic planning^5^ and liaisons with external public health, community coalitions, and emergency management collaborators.

The increasing frequency of outbreaks and epidemics of emerging and reemerging infectious diseases in the last decade has emphasized the significance of healthcare emergency preparedness programs, but the COVID-19 pandemic has tested healthcare facilities’ emergency plans and exposed vulnerabilities in healthcare emergency preparedness on a scale unexperienced in recent history.^6^ In addition, the clearly disparate impact of COVID-19 on vulnerable populations and communities has drawn attention to the need to better mitigate, plan for, and respond to health inequalities in healthcare emergency preparedness programs. We review the 4 phases of emergency management and explore the lessons to be learned from recent events in enhancing health systems capabilities and capacities to mitigate, prepare for, respond to, and recover from biological threats or events, whether it be a pandemic or a single case of an unknown infectious disease.

## Mitigation

**Fig. 1.**
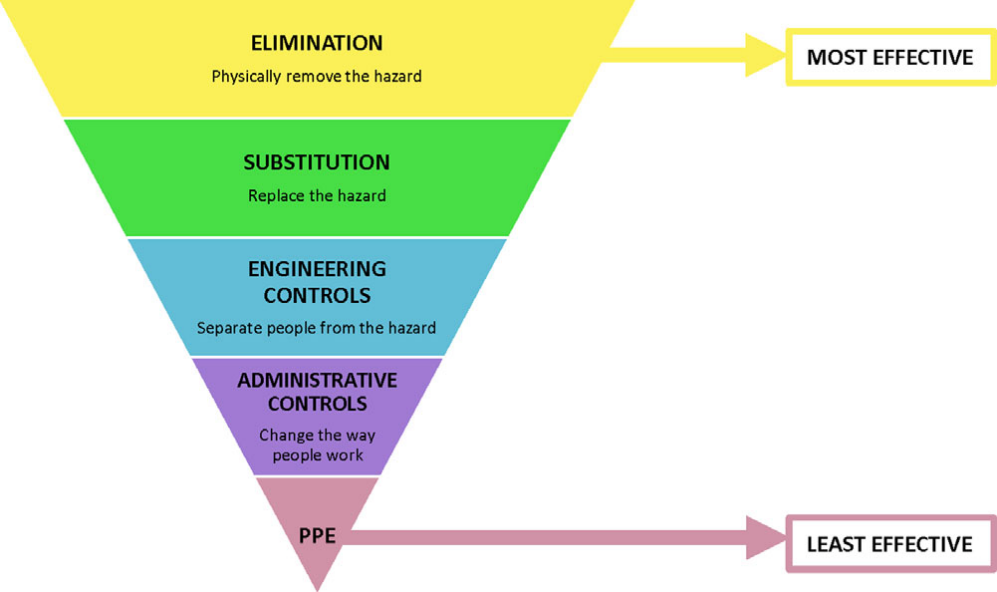
Hierarchy of controls. Adapted from the National Institute of Occupational Safety and Health (NIOSH) Hierarchy of Controls. Available at https://www.cdc.gov/niosh/topics/hierarchy/default.html. Not. PPE, personal protective equipment.

Mitigation activities prevent or reduce the likelihood of a disaster occurring or the impact and severity of the event. The first step to implementing the 4 phases of emergency management is an assessment of a facility’s vulnerabilities to potential hazards and threats and the direct and indirect effects the hazards may have on the hospital’s ability to provide patient services. The hazard vulnerability analysis (HVA)^7^ in healthcare enables cross-comparison of dissimilar hazards (eg, earthquake, terrorist attack, industrial accident, and outbreak), helps prioritize issues for emergency preparedness programs to address, and allows planning to be tailored to identified hazards with the greatest probability of affecting facility operations.^8^ With a regulatory requirement to be reviewed annually, a hospital’s HVA serves as a benchmark to measure changes and improvements in preparedness levels.^9^ Related to biological threats and events, countless mitigation activities can be implemented well in advance; the priorities identified through the HVA should guide facility-specific mitigation activities. The National Institute for Occupational Safety and Health (NIOSH) Hierarchy of Controls is also a helpful tool with which to prioritize and frame risk mitigation activities (Fig. 1);^10^ indeed, many of the COVID-19 mitigation guidelines released by the Centers for Disease Control and Prevention (CDC) were framed around the Hierarchy of Controls.

A core mitigation strategy for hospitals includes development of business continuity plans for maintaining operations during an emergency event, with an underlying premise to plan for the worst-case scenario. For an infectious disease emergency, this includes absence of an adequate workforce and resource and supply limitations. A business continuity plan must include identification of critical functions and staff, nonessential activities that can be suspended, and alternative strategies for resource allocation and supply chain access. These plans should also address impact to critical infrastructure, such as redundancy of power, communications, and data management systems. Although biological threats may not directly affect these elements, the COVID-19 pandemic highlighted the need for healthcare systems to enhance facility infrastructure to provide additional negative pressure rooms for isolation and technology infrastructure to enable remote work capabilities and a comprehensive telemedicine platform.

Certain disasters may be completely unanticipated, for which disaster-specific mitigation measures are either absent or of low priority (eg, hurricane mitigation in an area where hurricanes have historically been nonexistent). A global pandemic of a novel respiratory virus, however, has long been predicted, with only the precise timing and location of its emergence uncertain.^11^ Likewise, outbreaks and encounters with new HCIDs are increasingly occurring^12^ and numerous health systems across the US have experienced the presentation of a suspected case of Ebola virus disease (EVD), Middle East respiratory syndrome (MERS), or other HCID in their emergency department in recent years. Despite this inevitably, the COVID-19 pandemic highlighted gaps in health system mitigation for pandemic events and the unequal impacts of these events on different communities and populations. Even amid the ongoing COVID-19 pandemic, now is the time for hospitals to assess, refine, and implement mitigation measures for future pandemic threats and to redouble their efforts to identify especially vulnerable populations, anticipate their needs, and improve their abilities to respond to those needs.

## Preparedness

Hospital preparedness strategies improve organizational capability and capacity to respond to an emergency and are critical to local and national response to biological threats. Preparedness includes planning, training, and exercising for events that cannot be mitigated. Recent HCID outbreaks and the COVID-19 pandemic have underscored the need for hospitals to have well-developed and exercised standard operating procedures (SOPs), a trained workforce, communication plans, understanding of anticipatable needs of their vulnerable populations, clearly defined surge plans, and robust plans for supply chain management and staffing in the event of a biological threat.

### Planning

Healthcare facility preparedness should be defined by an emergency operations plan (EOP) that drives facility operations during an emergency. For biological events, the EOP should include considerations ranging from how to safely manage singular cases of suspected HCIDs that may present at a healthcare system to pandemic planning policies for large-scale events. Tools and protocols for the former include early screening and identification of suspect cases, designation of appropriate isolation areas in all frontline clinical spaces, and development of SOPs for internal workflows and informing appropriate external stakeholders. This framework, referred to as “identify, isolate, and inform,” was developed by the CDC for EVD but is adaptable to any HCID in any frontline clinical setting.^13,14^ Inadequate or delayed implementation of this framework has previously resulted in nosocomial transmission of HCIDs in hospital settings, as well as increased community spread of illness.^15,16^

Emergency management plans for presentation of suspected HCID patients should include the following: provisions for active screening at the initial clinical points of contact; indications for mobilization of appropriate resources, including personal protective equipment (PPE); immediate isolation of patient in an area identified well in advance; minimization of hospital operation disruptions; timely notification of internal and external stakeholders; well-trained staff; just-in-time training plans; and plans to ensure the patient receives medical evaluation and immediate care while conducting the appropriate diagnostic testing in parallel.^8^ A planning team that consists of physicians, infection preventionists, nurses, hospital epidemiologists, emergency coordinators, laboratory leadership, and facilities personnel should drive the development and refinement of these processes. Due to the high-risk setting that is HCID care, clinical decision making on invasive medical procedures to be offered should be considered well in advance and incorporated into hospital preparedness plans.^17^

In contrast to these sporadic suspected cases of a HCID, epidemic and pandemic planning require substantial efforts in anticipation of prolonged increased volumes of patients, the need to be able to expand isolation capabilities in care sites, and the potential for utilizing alternative care sites, as well as adaptability in scaling up and adjusting plans to changing resource access and rapidly evolving recommendations. These plans require careful considerations to maximizing available spaces, healthcare personnel, supplies and devices, and other resources in surge events. Epidemic planning is necessary for local outbreaks that require multiple simultaneous hospitalizations, and pandemic planning is built on the assumption that the incident is not geographically contained and that regional and federal capabilities and assets that would support other mass casualty events may not be readily available.

In 2017, the CDC published an accompanying checklist to the National Pandemic Preparedness Plan that included hospital-specific actions to guide pandemic planning.^18,19^ Although guidance and recommendations informed by previous outbreaks and disasters have been issued for healthcare surge capacity planning and resource allocation for pandemic events, specific recommendations for adequately addressing multiple waves of a continuing pandemic were generally lacking^20^ and must be incorporated into future hospital emergency preparedness plans and policies.

### Supply chain management

Over the past 2 decades, hospitals have generally evolved to function with a “just-in-time” approach to the supply chain, where few supplies are warehoused on site, but instead are delivered regularly as they are used. The vulnerabilities in that approach had national and international implications during the COVID-19 pandemic, leading to shortages of medical supplies and devices and deviation from accepted standards for respiratory protection and use of other PPE. National surveys of US nurses during the early months of 2020 found that <30% of employers had sufficient PPE to respond to a surge of patients^21^ and 87% reported having to reuse a single-use disposable respirator or mask with a patient infected with SARS-CoV-2.^22^ The Strategic National Stockpile stores supplies that can be administered nationwide within 12 hours; however, the supply is intended to supplement local resources and was not sufficient to address national needs seen during COVID-19 response.^23,24^

Healthcare systems must create supply caches, regularly review stockpile levels, and rotate supplies to ensure they are ready for use. For far-reaching events that exhaust national supply stocks, healthcare systems should include supply chain management plans in their EOP for rationing, allocating, and when necessary and appropriate reusing PPE and other supplies during a shortage. Coordination with local stakeholders, including public health, healthcare coalitions, and emergency management agencies, can help facilitate quicker access to available resources. Although the COVID-19 pandemic exposed significant gaps in all levels of supply chain management, future emergencies could affect supply chain issues that were virtually untouched during this pandemic, including waste services and gas or other fuel supply. It is important that as facilities retool their emergency plans for resource management, these elements are not overlooked.

### Facility management

A surge situation may warrant evaluation of facility management and changes in existing spaces and workflows, to include an inventory of isolation capabilities available for surge. Facility management may also include the establishment of alternate care sites in nontraditional settings within or outside of the facility. Screening and separation of patients suspected of having an infectious disease until they are confirmed or ruled out is a core management strategy to protect facility personnel and other patients. The significance of virtual platforms that can facilitate triage, including telemedicine and virtual screening applications, have emerged in the COVID-19 pandemic as key elements in routing suspected cases to designated entry points and directly to isolation areas rather than presenting in frontline clinical areas, reducing the risk of nosocomial pathogen transmission. Healthcare epidemiologists and infection preventionists are primed to lead these efforts in coordination with facility managers.

### Staffing and training

A pandemic planning assumption is that ˜40% of the workforce may be absent during a pandemic response.^18^ Staffing models will evolve during different phases of surge capacity situations, and emergency management plans should be flexible and account for changing situations. Staff providing direct patient care and management may be pared down to only the most essential to minimize staff exposure risks, with personnel being cross trained to perform other services outside of their typical responsibilities (eg, nurses performing environmental cleaning and disinfection). As the situation evolves (eg, patient volume increases, elective procedures are cancelled, high workforce absenteeism), personnel may be relocated to other wards and take on other duties. Staffing strategies used during the COVID-19 response include training nurses to a higher level of care, administrators returning to the bedside as nurse extenders, and team nursing.^25–27^

Alternative staffing models will inevitably warrant additional support for those redeployed, including training on standard operating procedures (SOPs), PPE, and other infection prevention and control elements. Likewise, adequate preparedness for presentation of a patient with an unknown infectious disease requires early identification and rapid implementation of infection prevention and control measures. To adequately prepare health systems for biological events across the spectrum of potential incidences (from a singular HCID suspected case to a pandemic), there is a need for ongoing assessment to ensure proficient infection prevention principles for all healthcare staff well before an emergency occurs. This not only protects the healthcare provider but can minimize transmission between patients and other staff.

### Testing and evaluating plans

Too often in emergency management, plans are developed, agreed upon, written into policy, and then stored on shelves until a regulatory requirement or emergency motivate their use. Instead, plans should be living documents, with regular exercising, revising, and updating to align with evolving practices and operations. Drills and exercises should be conducted with both internal and external partners and offer hospitals opportunities to test their plans based off realistic scenarios, including presentation of a HCID case in the emergency department, a surge situation stemming from a pandemic, or a natural or manmade disaster.

Low- to no-notice exercises^28^ are an especially effective way of evaluating preparedness plans and response capabilities as they are most reflective of real-life events. After-action reports and improvement plans, which identify strengths and gaps identified through the exercise and trackable corrective actions to address gaps, are vital to improving hospital preparedness; however, more importantly, plans should be developed to reevaluate updated protocols at regular intervals to ensure revisions were successful in a continuous cycle of improvement.

## Response

Response activities are aimed at reducing mortality and morbidity of a disaster, limiting overall socioeconomic costs, and maintaining essential hospital services. A facility’s EOP should guide their response to an infectious disease emergency, leveraging lessons learned from the preparedness phase to optimize response activation and effectiveness.^9^ Frequent communication and coordination with external stakeholders, particularly other local hospitals and public health agencies, is critical to ensure rapid dissemination of information and unified responses, particularly in events with novel pathogens where recommendations frequently evolve as more is known about the causative agent. The establishment of a medical emergency operations center, or a similar structure, can facilitate cross-healthcare coalition collaboration and enhance coordination of the use of healthcare capacity across a city or region when resources are becoming overloaded.^29^ Equally important is utilization of an internal platform to quickly push out updated guidance, recommendations, SOPs, training materials, and other information to staff during a biological event. Hospital intranet sites provide a “one-stop shop” for the most recent and relevant information during a disaster response.

A fundamental component of health system response to an emergency includes mobilization of the Hospital Incident Command System (HICS), which is outlined in a hospital’s EOP. As a scalable system built on an all-hazards approach to manage operations, information flow, and logistics in a systematic manner, HICS can be tailored to specific events. The HICS outlines key positions and responsibilities during an emergency event, including command staff (a medical-technical specialist, a safety officer, a liaison officer, a public information officer) and general staff divided into sections for operations, planning, logistics, and finance.^8,30^ Hospital epidemiologists or an infectious diseases physician may be tapped during a biological event to serve as the medical-technical specialist to provide subject matter expertise to command staff and media. Infection preventionists are likely to serve as the safety officer to provide overall safety-related expertise to command staff and beyond. Medical leaders and/or capacity specialists are needed to ensure appropriate balancing and continual monitoring of capacity mobilization efforts with the need to provide essential medical care for the community.

The HICS system is well-suited for coordinating communication and resources during emergency events; however, the extensive and lengthy activation of HICS during the COVID-19 pandemic identified other needs to augment limitations of HICS. For example, the pandemic resulted in the need for incorporation of long-term changes to clinical practices that land outside the scope of the HICS.^31^ Parallel yet integrated processes for addressing hospital response needs not adequately consolidated in HICS should be considered in emergency management plans.

## Recovery

The recovery phase, which may begin amid the response phase, includes restoration efforts and resumption of normal, or ‘new normal’ operations and includes considerations for facility recovery, staff recovery, and financial recovery.^9^ Even though diverse stakeholders are generally unified during the response phase on a singular goal, recovery is complex and can be prolonged. During and following a major event, hospitals should compile data and lessons learned into an after-action report and improvement plan that outlines corrective actions and revisions to emergency management programs.^32^ A priority in the recovery phase for healthcare systems is the well-being of personnel, which has been reinforced during the COVID-19 pandemic. Anxiety, fear, and pressure stemming from high-risk situations, even among highly trained and experienced clinical teams, should be anticipated, as should the potential for negative reactions from community members and even family and friends. Psychological and specialist support should be easily accessible and highly encouraged as staff respond to and recover from emergencies.

## Other facilities

One of the most significant learnings from the COVID-19 pandemic will undoubtedly be the need for robust emergency preparedness program implementation in all healthcare settings, including long-term care facilities, skilled nursing facilities, outpatient clinics, urgent care centers, home health agencies, and other specialized centers such as surgical and dialysis centers. No healthcare setting was spared by the pandemic; indeed, some of these settings were epicenters of the most severe outbreaks of COVID-19 due to their vulnerable populations. Long-term care and skilled nursing facilities, in particular, were severely impacted, and constraints in the long-term care system availability negatively affected acute care hospital throughput and capacity.^33^ The vulnerability of these settings was well known: long-term care facility residents have historically reported increased severe outcomes (eg, hospitalizations, death) following disasters,^34,35^ and routine seasonal outbreaks of influenza in these settings are a significant challenge.^36^ There is an obligation among healthcare, public health, and emergency management communities to improve emergency preparedness programs and training in these other essential healthcare settings to ensure the toll experienced during the COVID-19 pandemic is not repeated in future infectious disease emergencies.

In conclusion, a pandemic on the scale of COVID-19 was not unpredicted but was widely underestimated. The increasing frequency of HCID cases, emergence of new pathogens, and the COVID-19 pandemic emphasize the elevated and ongoing threat of biological hazards to healthcare systems. A recurring cycle of assessing, planning, training, exercising, and revising is vital to maintaining healthcare system preparedness, even in absence of an immediate, high-probability threat. These tasks have been historically challenging due to gaps in activities needed for robust preparedness and the resources and funding made available to do so.^37^ Healthcare epidemiologists and infection preventionists must play a pivotal role in incorporating lessons learned from the pandemic into emergency preparedness programs and building more robust preparedness plans.

Facilities should waste no time in using local, regional and national lessons learned from the COVID-19 pandemic to reevaluate and shore up known gaps, drive re-review and revision of existing mitigation strategies and epidemic and pandemic preparedness plans, and implement innovations emanating from the COVID-19 response. However, areas that went untested during the pandemic should not be overlooked, such as how to optimally and safely identify, isolate, and manage a suspected HCID patient presenting to an emergency department or clinic. Although the world’s focus has recently been centered on COVID-19, 2 independent outbreaks of EVD in sub-Saharan Africa, ongoing cases of MERS in the Middle East, and recent imported cases of monkeypox in travelers to the United Kingdom and the United States are a reminder for healthcare systems to remain vigilant and prepared for all biological threats.
